# Ion-Movement-Based Synaptic Device for Brain-Inspired Computing

**DOI:** 10.3390/nano12101728

**Published:** 2022-05-18

**Authors:** Chansoo Yoon, Gwangtaek Oh, Bae Ho Park

**Affiliations:** Division of Quantum Phases & Devices, Department of Physics, Konkuk University, Seoul 05029, Korea; jl30124@konkuk.ac.kr (C.Y.); gwang@konkuk.ac.kr (G.O.)

**Keywords:** ion movement, synaptic device, brain-inspired computing

## Abstract

As the amount of data has grown exponentially with the advent of artificial intelligence and the Internet of Things, computing systems with high energy efficiency, high scalability, and high processing speed are urgently required. Unlike traditional digital computing, which suffers from the von Neumann bottleneck, brain-inspired computing can provide efficient, parallel, and low-power computation based on analog changes in synaptic connections between neurons. Synapse nodes in brain-inspired computing have been typically implemented with dozens of silicon transistors, which is an energy-intensive and non-scalable approach. Ion-movement-based synaptic devices for brain-inspired computing have attracted increasing attention for mimicking the performance of the biological synapse in the human brain due to their low area and low energy costs. This paper discusses the recent development of ion-movement-based synaptic devices for hardware implementation of brain-inspired computing and their principles of operation. From the perspective of the device-level requirements for brain-inspired computing, we address the advantages, challenges, and future prospects associated with different types of ion-movement-based synaptic devices.

## 1. Introduction

Explosively increasing data set sizes in artificial intelligence (AI) and the Internet of Things have resulted in us facing limitations in energy efficiency and the challenges of the von Neumann bottleneck approaching the end of Moore’s law [1,2,3,4,5]. In more detail, the AI algorithm that runs on the complementary metal oxide semiconductor (CMOS)-based von Neumann hardware seems to be somewhat inefficient [6], since: (1) the separation of processor and memory causes heavy data traffic between devices, especially in data-intensive tasks; (2) CMOS uses a “0” and “1” binary logic system rather than a gradual weight change; (3) the connections of transistors in silicon chips are usually in two dimensions; (4) CMOS-based computers consume a thousand times as much energy as the human brain to perform the same task [6,7]. For example, a supercomputer (IBM Watson) [8] has 2880 computing cores (10 refrigerators’ worth in size and space) and requires about 80 kW of power and 20 tons of air-conditioned cooling capacity, while the human brain occupies a space of less than 2 *l* and consumes low power, of the order of 10 W [9,10]. Additionally, if data storage and communication continue to increase at the current rate, the total energy consumed by binary operations using CMOS will reach ~10^27^ J in 2040, surpassing the total energy produced worldwide [11,12]. In order to improve the performance of computing systems in the so-called “big data” era [13], it is necessary to fundamentally change the way computing is executed [14]. We need to shift to a data-centric paradigm rather than a computer-centric one [14].

The growth of computational power, the availability of big data, and the rapid development of training methods have resulted in significant advances in data-centric computing methods, such as the artificial neural network (ANN), which is inspired by the co-location of logic and memory, tolerance to local failures, hyper-connectivity, and parallel processing present in the human brain [12,15,16]. The human brain is a massively parallel computing structure that processes input information by synaptic transmission. Each synaptic event consumes only around 1–10 fJ [5,17]. The hardware in brain-inspired computing architecture is required to mimic stochastic behaviors by introducing a new logic device such as an orthogonator gate and to physically emulate synapses in the human brain at the small circuit or device levels, consuming significantly reduced energy [12,18,19,20,21,22,23,24].

Recently, synaptic devices based on various types of resistive switching devices have been utilized for brain-inspired computing [25,26,27,28,29,30]. Brain-inspired computing based on synaptic devices has achieved particular progress from ion-movement-based resistive switching mechanisms such as cation-movement-based filaments [31,32,33,34,35,36], anion-movement-based filaments [37,38,39,40,41,42,43], cation-movement-based ferroelectric polarization reversal [44,45,46,47,48,49], and ion-movement-based electrochemical electrolytes [50,51,52,53,54,55,56,57]. These ion-movement-based resistive switching devices can show a gradual change in conductance and nonvolatile characteristics, which have not been implemented in Mott-insulator-based resistive switching devices. Resistive switching based on the phase transition of Mott insulators under an electric field shows an abrupt change in conductance and volatile behaviors, which are suitable for neuron devices [58,59,60]. In this review, we summarize in detail the different ion-movement-based mechanisms and discuss the advances in the development of ion-movement-based resistive switching devices for artificial synaptic elements. In addition, we discuss the challenges that need to be addressed in future research on synaptic devices towards developing brain-inspired computing systems.

## 2. Biological Synapses

### 2.1. Properties of Biological Synapses

**Figure 1:**
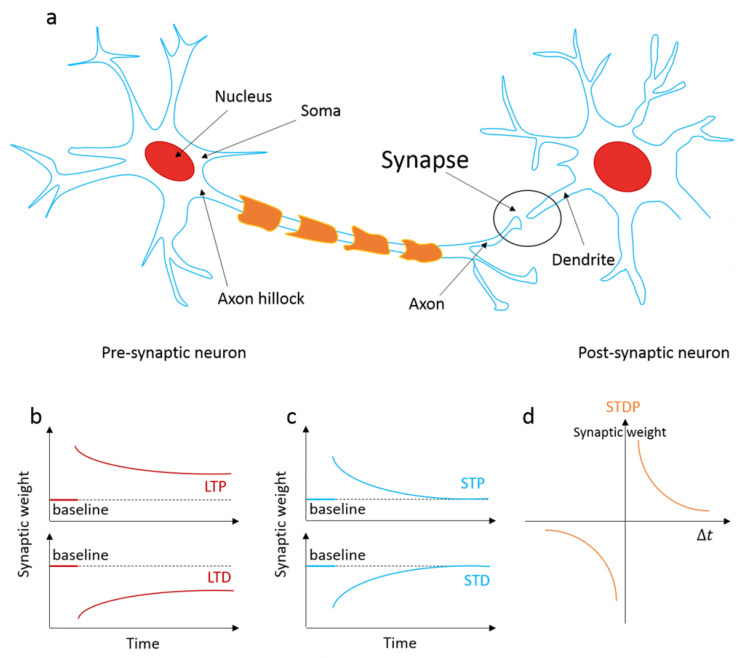
Properties of a biological synapse. (**a**) Schematic image of the biological synapse structure. Typical synaptic weight changes in the cases of (**b**) LTP and LTD, (**c**) STP and STD, and (**d**) STDP.

The brain contains billions of neurons, which are highly connected by trillions of synapses [6]. Synapses are small gaps (20–40 nm) between the axon end of the presynaptic neuron and the dendrites of the postsynaptic neuron (Figure 1a) [61]. Neurons generate action potentials (spikes) with amplitudes of approximately 100 mV and durations in the range of 0.1–1 ms in their soma. The spikes propagate through the axon and are transmitted to the postsynaptic neurons through the synapses [10]. The connection weights of the synapses between neurons can become stronger (potentiation) or weaker (depression) through a process called synaptic plasticity, as the brain adapts to new information. Synaptic plasticity is widely believed to play a key role in the learning and memory processes of the brain [61].

### 2.2. Biological Synaptic Plasticity

Biological synaptic plasticity, which enables learning and memory, is the ability to continuously modulate synaptic weights in response to action potentials. In more detail, biological synaptic plasticity is the changed connection strength of a biological synapse, which is caused by the activities of a presynaptic neuron and a postsynaptic neuron. [10] It includes long-term potentiation/depression, short-term potentiation/depression, and spike-timing-dependent plasticity (STDP). The biological synaptic weight can be persistently changed by modulating the amount of neurotransmitter presynaptically released across the synapse or the number of receptors present postsynaptically; this is so-called long-term plasticity. Specifically, the increases and decreases in synaptic weight are called long-term potentiation (LTP) and long-term depression (LTD), respectively (Figure 1b) [5,62]. Short-term potentiation (STP) and short-term depression (STD) are temporary increases and decreases in synaptic weight, respectively. STP and STD generally last from seconds to tens of minutes and then fade away to the initial values, in biological systems (Figure 1c) [5,63]. Spike-timing-dependent plasticity (STDP), a form of Hebbian learning, emerged as a new concept of cellular learning in the late 1990s [10,64,65,66]. Different types of STDP exhibit different forms of dependency on the spiking time $\Delta t=t_{pre}-t_{post}$, where $t_{pre}$ and $t_{post}$ are the arrival times of presynaptic and postsynaptic spikes, respectively. As the amplitude of Δt becomes shorter, the change in synaptic weight becomes larger (Figure 1d).

## 3. Resistive Switching Devices Applicable to Synaptic Devices

### 3.1. Synaptic Devices for ANNs

#### 3.1.1. Brain-Inspired Computing with ANNs

**Figure 2:**
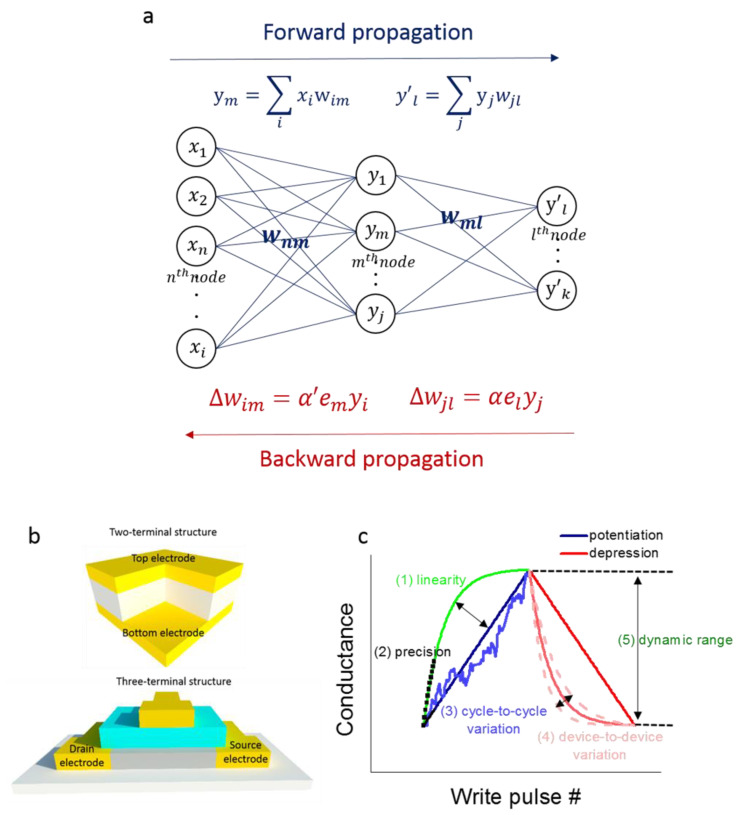
(**a**) Schematic structure of fully connected ANN. (**b**) Schematic structure of two-terminal- and three-terminal-based synaptic devices. (**c**) An overview of functional metrics of analog resistive switching for improved learning capability.

The implementation of brain-inspired computing with ANNs could overcome the challenge for software-based computing where the algorithm is essentially run by conventional sequential machines with limited parallelism [29]. An ANN generally consists of a massive number of synapses that connect between groups of neurons, which requires a large volume of memory and thus results in extremely high hardware costs. Therefore, it is beneficial to develop more compact synaptic devices with high performance [67]. Figure 2a shows the vector matrix multiplication (VMM) required for typical ANN learning. The processing of an ANN architecture includes two phases: forward and backward propagation. First, the input data x are unwrapped to a row vector (1×n), and each input datum is connected to each value (weight w) of the next layer, which is arranged in a matrix for VMM. During forward propagation, the VMM $y_{m}=\underset{n}{\sum }w_{nm}x_{n}$ is required and can be calculated by $I_{m}=\underset{n}{\sum }G_{nm}V_{n}$ in a crossbar array of synaptic devices, where $G_{nm}$ is the weight (conductance) of the synaptic devices at node (n, m) and $V_{n}$ is the input voltage of row n. During backward propagation, the weight of the synaptic device is modulated to reduce the gap between the real output and the target output. The error at the jth node is calculated as $e_{j}=t_{j}-y_{j}$, where $y_{j}$ is the real output and $t_{j}$ is the target output. In addition, $e_{j}$ is used to guide the adjustment of the weight, as $\Delta w_{ij}=\alpha e_{j}x_{i}$, where α>0 is a constant learning rate and $x_{i}$ is the ith input. Synaptic devices must have controllable multistate and analog conductance for processing a huge amount of information like a biological brain [5]. The other method is brain-inspired computing with a spiking neural network (SNN), which consists of STDP-based learning rules. In SNN, the biological system is more closely mimicked than in ANN. SNN is believed to be computationally powerful. However, SNN has still not been able to reach the accuracy of ANN trained with backpropagation, due to the lack of efficient learning algorithms [68]. Therefore, suitable STDP-based learning rules for the improved accuracy of SNN must form the subject of further research. Consequently, this review focuses on ANNs with synaptic devices, which are relatively far from real synapses and brain mimicking but use the power of parallel inference to drastically reduce the energy requirements for hardware operating with a large neural network [68].

#### 3.1.2. Synaptic Devices

Many studies have attempted to implement synaptic devices emulating the brain’s synaptic plasticity and functions such as memory, learning, and recognition. Synaptic devices modulate their conductance values in a way that is analogous to the modulation of synaptic weights in biological systems. In synaptic devices, the conductance is generally measured using the current between two electrodes, as shown in Figure 2b. A synaptic device’s conductance is equivalent to the biological synaptic weight and can be increased or decreased by an external voltage signal leading to potentiation or depression characteristics, respectively. In analogous conductance behavior such as potentiation and depression in the synaptic device, the functional parameters should be checked with respect to the goal of reducing accuracy loss in the ANN. The accuracy loss in ANNs in the implementation of a brain-inspired computing system is a critical factor for quantifying the reliability metrics and can be attributed to two main factors: the nonideal effect in analog resistive switching, which causes the hardware training accuracy to be lower than the software training accuracy [68,69], and the conductance drift or fluctuation of the analog resistive switching devices, which causes the accuracy after training to degrade with time [68,70].

### 3.2. Required Specifications of a Synaptic Device for ANNs

The functional metrics of analog resistive switching behavior corresponding to linearity, precision, cycle-to-cycle variation, device-to-device variation, and dynamic range have direct influences on the training accuracy in brain-inspired computing, as shown in Figure 2c. The negative effect or error in the training accuracy shown by the degradation of these functional metrics during repeated operations should be taken into careful consideration. Many simulation studies have reported how much these functional metrics impact on the training accuracy in ANNs. Based on previous valuable simulation studies, we discuss the definitions of functional metrics and their impacts on training accuracy.

#### 3.2.1. Linearity

The linearity of weight updates represents how linearly the conductance changes with the number of applied pulses. Ideally, the amount of potentiation and depression should be linearly proportional to the number of applied pulses [71]. Lower linearity of potentiation (depression) results in greater difficulty in tuning the conductance to the target value, leading to worse convergence rates during training [67,68,70]. Hence, higher nonlinearity directly causes more accuracy loss. However, the dependence of training accuracy on linearity changes with the type of learning algorithm. For learning with backpropagation algorithms, the linearity of the weight update dramatically affects the online training accuracy. High accuracy can only be achieved with small nonlinearity [72]. In contrast, on-chip learning with sparse coding algorithms demonstrates only a small accuracy loss of ~4%, even at the maximum nonlinearity [67,73]. This implies that linearity has a less critical impact on learning accuracy when sparse coding algorithms are used rather than backpropagation algorithms.

#### 3.2.2. Precision

Precision is defined as the number of conductance levels that the synaptic device can achieve [67,68,71]. High precision is known to bring advantages in terms of network capacity and robustness, although the requirement for the precision remains application-specific [10]. If the precision is smaller than 4 bits, the classification accuracy in a single-layer perceptron neural network with a compressed modified National Institute of Science and Technology (MNIST) data set decreases abruptly [74]. Even for sparse coding algorithms for on-chip learning and backpropagation algorithms with emerging nonvolatile memory, the learning accuracy decreases when the precision is smaller than 6 bits [67,72].

#### 3.2.3. Cycle-to-Cycle Variation

In the potentiation and depression process, hardware systems based on resistive switching devices suffer from random noise addition. The cycle-to-cycle variation is defined as pulse-to-pulse noise at every weight update operation in the synaptic device during potentiation or depression. Cycle-to-cycle variation can cause unregulated changes in the conductance after applying each pulse. Due to the large number of weight update operations during training, cycle-to-cycle variation results in increased cost of training and serious accuracy loss [67,68,75].

#### 3.2.4. Device-to-Device Variation

The effect of device-to-device variation can be analyzed by introducing variation in the linearity baseline for each synaptic device. This functional metric has a smaller impact on learning accuracy than other functional metrics. On-chip learning with a sparse coding algorithm can tolerate a device-to-device variation of up to 30% [67,73]. In the case of nonvolatile memory-based synaptic devices with a backpropagation algorithm, the neural network is resilient to device-to-device variation, except where there is high nonlinearity in the weight update [72].

#### 3.2.5. Dynamic Range (Analog on/off Ratio)

The dynamic range, also known as the analog on/off ratio, can be defined as the ratio of the high resistive state (HRS) conductance and the low resistive state (LRS) conductance in analog switching. The dynamic range varies with the materials and structures of analog resistive switching devices. The learning accuracy for the synaptic device dramatically decreases when the dynamic range becomes smaller [67,73,76]. In addition, a large dynamic range in a synaptic device results in improved tolerance to cycle-to-cycle and device-to-device variation [63].

### 3.3. Ion-Movement-Based Mechanisms of Different Resistive Switching Devices

#### 3.3.1. Cation-Movement-Based Filamentary Two-Terminal Resistive Switching Devices

Resistive switching devices based on cation migration are usually termed electrochemical metallization (ECM) cells or conductive-bridge random-access memories (CBRAMs) in the literature [77,78,79,80,81]. The ECM cell is a promising approach for brain-inspired computing devices due to its short switching time (~ns), scalability to the nanometer regime, and ultra-low power consumption (~nW) [82]. A typical ECM cell utilizes an insulator as a switching layer sandwiched between the top and bottom electrodes. One of the two electrodes should be an electrochemically active metal (e.g., Ag, Cu, or Ni) and the other should be an inert metal (e.g., Pt, W, or Au) [78,81,83,84]. The insulator layer acts as a solid electrolyte for Ag, Cu, or Ni cation migration. Insulator layer materials include chalcogenide glasses [85], oxide layers [86], epitaxy layers [35], polymers [34], and even two-dimensional (2D) materials [31].

**Figure 3:**
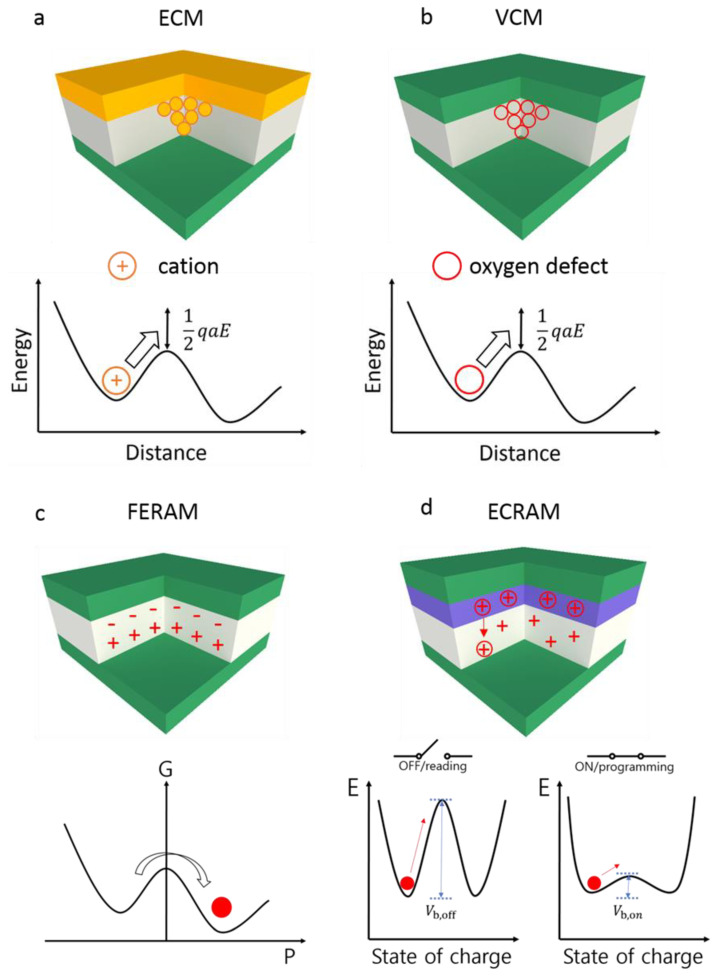
Ion-movement-based resistive switching devices. (**a**) Schematic of structure and of the field-driven acceleration of cation transport for ECM-based resistive switching device. (**b**) Schematic of structure and of the field-driven acceleration of defect transport for VCM-based resistive switching device. (**c**) Schematic of structure and of the field-driven cation movement for FERAM-based resistive switching device. G and P denote Gibbs energy and polarization, respectively. (**d**) Schematic of structure and of operation-specific change in blocking barrier for ECRAM-based resistive switching device. E denotes energy barrier.

During resistive switching, essentially new materials are produced in the insulator layer through electrochemical reactions and ion movement. When a positive bias is applied to the active electrode, three steps are normally involved in this material reconfiguration process called set switching: ionization, movement, and the reduction of cations to form conductive filaments (CFs) [77,79,81,84,87]. The active metal used as an anode is ionized to a cation under an external voltage bias (Figure 3a) [77,88,89]. The cation moved by the applied electric field travels through the solid electrolyte toward the inactive cathode electrode. The speed of ion movement depends exponentially on the applied electric field due to the barrier lowering effect, as shown in Equation (1) [77,81,90].

*v*_c_ = *a∙ f ∙* exp (− *E*_a_/*k*_B_*T*) *∙* sinh (*qaE*/2*k*_B_*T*) (1)

where *v*_c_ is the drift velocity of the cation, *a* is the hopping distance (the distance between the potential wells), *f* is the attempt frequency, *E*_a_ is the energy barrier for ion migration, *E* is the applied electric field, *k*_B_ is Boltzmann’s constant, *q* is the ion’s charge, and *T* is the absolute temperature. Electrocrystallization is caused by the reduction of cations with the aid of electrons provided by the inert metal cathode, leading to the formation of CFs [79,81,83,84]. On the other hand, when a negative bias is applied to the active electrode, the formed CFs can be ruptured due to thermally assisted electrochemical reactions, and this is called reset switching [91]. The front of the CF has the thinnest cross-sectional area and hence the highest current density, leading to a high temperature. Under the negative voltage applied to the active electrode, which prevents the supply of cations, the dissolution reaction at the front of the CF will be accelerated by the high temperature [33]. In addition, the cation-movement-based mechanism should consider the time evolution of the memory characteristic after the set process. The memory characteristic degradation relies on the diffusion of metal atoms toward the lower concentration region over time [68].

#### 3.3.2. Anion-Movement-Based Filamentary Two-Terminal Resistive Switching Devices

An anion-movement-based filamentary two-terminal device, also known as valence change memory (VCM), is quite similar to a cation-movement-based filamentary two-terminal device (Figure 3b), except that the CF formed in the insulating thin film is a chain of oxygen defects within an oxide (rather than a chain of metallic atoms of the reactive electrode through an insulating solid electrolyte) [82]. When a positive bias is applied to the top electrode, anions hop among the energy potential wells (assumed to have hopping distance *a* and energy barrier *E*_a_), where the applied electric field *E* lowers the energy barrier by a factor of *qaE*. The anion drift velocity shows the same exponential dependence on *qaE* as that of the cation drift velocity presented in Equation (1) [39]. The anions drift toward the top electrode leading to the movement of oxygen defects to the bottom electrode and the growth of defect-based CFs. On the other hand, when a negative bias is applied to the top electrode, the formed defect-based CF ruptures from the top electrode. The anion-movement-based filamentary system is attractive because it requires only metal oxides such as HfO_x_, AlO_x_, WO_x_, FeO_x_, GdO_x_, TaO_x_, TiO_x_, and mixtures of such films, many of which are already used in CMOS processes. The underlying metal–insulator–metal structure is simple, compact, CMOS-compatible, and highly scalable, and its energy consumption and programming current can be made ultra-low, up to sub-pJ and <1 μA, respectively [82].

#### 3.3.3. Cation-Movement-Based Ferroelectric Two-Terminal Resistive Switching Devices

In a prototypical ferroelectric material, the charge centroids of positive and negative ions do not overlap. Ferroelectricity arises from the spontaneous displacement of the positively charged cations from the negatively charged oxygen anions in crystal structures such as ABO_3_-type perovskites [92]. Mesoscopic one-dimensional free energy potentials can model 180° switching processes with two energy wells and one local maximum between the two wells, as shown in Figure 3c. The model is based on a mesoscopic lattice element, which comprises a large number of microscopic lattice cells. The application of an electric field distorts the energy landscape, and a dipole switch occurs when the equilibrium value determining the B-ion position exceeds the unstable equilibrium due to the central O^2−^ ion [93]. As a consequence, by application of a sufficiently high external electrical field, the B ion can equivalently move towards the “top” or the “bottom” of the lattice, thereby leading to the “up” or “down” states, respectively. A permanent dipole moment resulting from B-ion movement can be maintained even in the absence of an external electric bias [92,93]. A ferroelectric material such as Pb(Zr,Ti)O_3_ [33], (Hf,Zr)O_2_ [94], BiFeO_3_ [45], or BaTiO_3_ [44,46,95] acts as an active layer in cation-movement-based ferroelectric two-terminal resistive switching devices, which are also known as ferroelectric random-access memory (FERAM).

#### 3.3.4. Ion-Movement-Based Electrochemical Three-Terminal Resistive Switching Devices

More recently, researchers proposed a cation-movement-based electrochemical transistor system, also called electrochemical random-access memory (ECRAM), comprising an ion-conducting/electron-blocking gate in combination with a channel layer (Figure 3d) [96]. The charge in the channel is manipulated during the “write” operation, which allows both electrons (through the external circuit) and ions (through the electrolyte) to be exchanged between the channel and the reference electrode under a small bias [97]. During the “read” operation, where the channel resistance is sensed, the circuit between the channel and the reference electrode is open, and the electronic charge of the channel remains unaltered by virtue of an ion-conducting/electron-blocking electrolyte [61,97]. Hence, a cation-movement-based electrochemical system is a type of nonvolatile redox cell in which the state of charge in the channel determines the electronic conductivity [61]. Typical materials systems used in ion-movement-based electrochemical three-terminal resistive switching devices are MoO_3_/ionic liquid [98], MoO_3_/LiClO4:PEO [62], SmNiO_3_/ionic liquid [99], graphene/LiClO_4_ [97], and MoS_2_/PTCDA [100]. Organic materials are widely used in ion-movement-based electrochemical devices, and thus they are also known as organic electrochemical transistors [61,62,63,101].

## 4. Synaptic Devices

### 4.1. Cation-Movement-Based Filamentary Two-Terminal Synaptic Devices

To mimic biological synaptic plasticity, it is essential to demonstrate repeatable analog switching with ultra-low power or energy consumption. The attractive characteristics of the ECM cell such as short switching time, scalability, and ultra-low power or energy consumption have driven the development of synaptic devices. To improve the performance of energy-efficient analog switching, emerging electrolytes for ECM cells such as 2D materials, nanowires, polymers, and ultra-thin films have been used [31,32,33,34,102].

**Figure 4:**
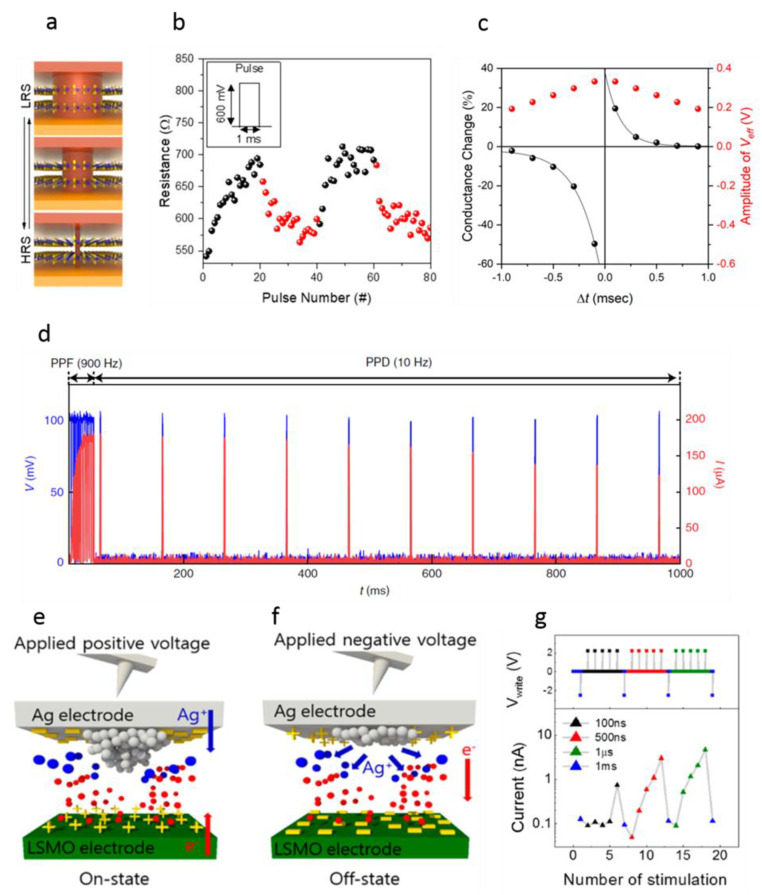
(**a**) Schematic of the change in the cation–movement–based filament for the Cu/MoS_2_ double-layer/Au with switching between HRS and LRS. (**b**) Potentiation–depression curve obtained by consecutive pulses with a small amplitude of 0.6 V and (**c**) STDP behaviors obtained by low–amplitude spiking for the Cu/MoS_2_ double–layer/Au. Reprinted/adapted with permission from Ref. [31]. 2019, American Chemical Society. (**d**) The current response of frequency–dependent PPF and PPD obtained by input pulses (100 mV, 1 ms) in a cation–movement–based filamentary two–terminal synaptic device with protein nanowires. Reprinted/adapted with permission from Ref. [32]. 2020, Springer Nature. (**e**,**f**) Schematic illustrations of growth and rupture of an uncompleted CF of Ag in an Ag/PZT/LSMO device in LRS and HRS, respectively. (**g**) Potentiation behaviors obtained by stimulation pulses with different durations for an Ag/PZT/LSMO device. Reprinted/adapted with permission from Ref. [33]. 2017, American Chemical Society.

The Cu/MoS_2_ bilayer/Au system has been reported for a synaptic device with ultra-low switching voltage (Figure 4a,b) [31]. In the MoS_2_ bilayer between an active Cu top electrode and an inert Au bottom electrode, Cu has a lower migration barrier and diffusion activation energy than sulfur vacancies, leading to the operation of the synaptic device at ultra-low switching voltages. A pulse with a small amplitude of 0.6 V can cause a gradual increase and decrease in the device resistance, resulting in potentiation–depression curves (Figure 4b). Such low switching voltages are important, especially for neuromorphic computing, which strives to operate with a power far lower than that required for digital computing [31]. In addition, the STDP of the cation-movement-based filamentary two-terminal synaptic device was experimentally confirmed, as shown in Figure 4c. The developed device reveals consistent analog switching and thus exhibits synapse-like learning behavior such as STDP, which was demonstrated for the first time in 2D-material-based vertical memristors. This demonstration of STDP combined with low switching voltage is promising for applications in neuromorphic circuits.

Fu et al. demonstrated a type of diffusive memristor which is fabricated using protein nanowires harvested from the bacterium Geobacter sulfurreducens and operates at biological voltages of 40–100 mV [32]. They demonstrated that a reduction in the switching voltage is obtained by using protein nanowires. The influx and efflux of Ag in the CF can also emulate Ca^2+^ influx and extrusion in a biological synapse. The steady-state evolution of the CF and the resultant change in conductance can be used to emulate synaptic plasticity. Figure 4d shows frequency-dependent paired-pulse facilitation (PPF) and paired-pulse depression (PPD) in a synaptic device operating at biological voltages. The features of the protein-nanowire-based filamentary devices are very similar to those of biological synapses in terms of signal amplitude and/or frequency range.

It has been reported that a synaptic Ag/PbZr_0.52_Ti_0.48_O_3_ (PZT)/La_0.8_Sr_0.2_MnO_3_ (LSMO) device with an ultra-thin ferroelectric PZT layer (~4 nm) serving as an electrolyte for cation migration can achieve very low energy consumption [33]. The ferroelectric barrier width becomes thinner due to the growth of an uncompleted CF of Ag, leading to a higher tunneling transmittance and switching to a low on-state resistance, as shown in Figure 4e. The rupture of the Ag CF takes place due to a thermally assisted electrochemical reaction (Figure 4f). Then, the tunneling barrier width becomes thicker, and the device switches back to a high off-state resistance. The gradual change in the direct tunneling current of the Ag/PZT/LSMO is enabled by barrier-width control originating from the Ag ion migration. Both potentiation and depression can be induced in the Ag/PZT/LSMO device by short stimulation pulses with a duration of 100 ns, which are enabled by the combination of applied voltage and polarization bound charge, as shown in Figure 4g. This characteristic could result in low programming energy (a potentiation energy consumption of ~22 aJ and a depression energy consumption of ~2.5 pJ) by minimizing the programming time.

**Figure 5:**
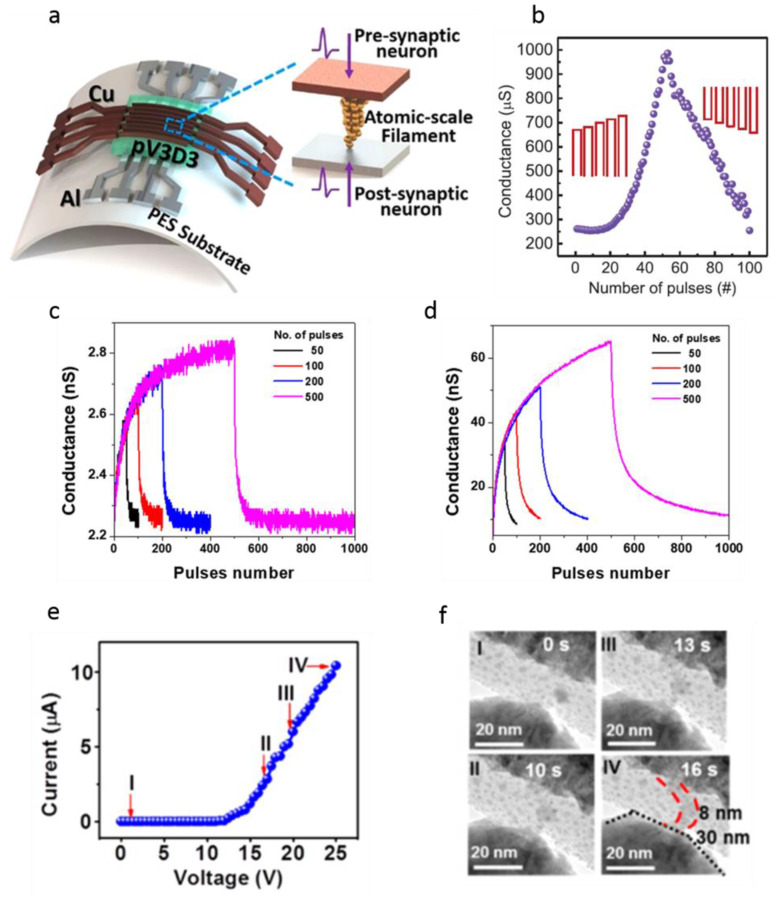
(**a**) Schematic image of pV3D3-based flexible synaptic device using the formation and rupture of Cu ion-movement-based filament. (**b**) Potentiation (depression) curve obtained by smart pulse scheme with increasing amplitude in the range of 2~4 V (−1.2~−1.4 V) with a width of 60 ns (100 ns) for a pV3D3-based flexible synaptic device. Reprinted/adapted with permission from Ref. [34]. 2018, American Chemical Society. (**c**,**d**) A comparison of potentiation–depression curves of the Ag/SiN_x_/a-Si/p^++^ Si-based devices with unimplanted (**c**) and implanted (**d**) devices under varying numbers of stimulation pulses of 7.0 (−3.0) V for potentiation (depression). Reprinted/adapted with permission from Ref. [36]. 2020, Springer Nature. (**e**) Gradual current change and (**f**) simultaneously obtained transmission electron microscope image of a Cu tip/SiO_2_/W cell during injection of voltage with increased amplitude. Reprinted/adapted with permission from Ref. [86]. 2017, American Chemical Society.

Jang et al. demonstrated that the transition of the operation mode in a poly(1,3,5-trivinyl-1,3,5-trimethyl cyclotrisiloxane) (pV3D3)-based flexible memristor from conventional binary switching to synaptic analog switching can be achieved simply by reducing the size of the formed filament [34]. The flexible pV3D3 memristor operates through the formation and rupture of the Cu CF inside the pV3D3, resulting in the LRS and HRS, respectively, as shown in Figure 5a. Potentiation and depression curves can be induced by the conductance updates of the pV3D3 memristor under the application of consecutive pulses, as shown in Figure 5b. Conductance updates with linear behavior are achieved by utilizing pulse trains with increasing amplitude in the range of 2~4 V (−1.2~−1.4 V) with a width of 60 ns (100 ns) for the potentiation (depression) process.

#### Challenges for Cation-Movement-Based Filamentary Two-Terminal Synaptic Devices

Reproducible gradual switching and controllable formation of the CF in cation-movement-based filamentary synaptic devices has been challenging to achieve due to the abrupt and stochastic nature of the switching mechanism, especially in the set process [61,82,103]. Therefore, a cation-movement-based filamentary two-terminal synaptic device typically shows nonlinear and asymmetric conductive responses, as well as large device variations, which can deteriorate the performance of an ANN [104,105]. The nonlinear and asymmetric conductivity can be overcome by introducing smart pulse schemes with varying amplitudes or durations of pulses, producing a large number of intermediate states and a symmetric/linear response [67,106]. However, smart pulse schemes with non-identical pulses require a read-before-write step to first identify the conductance state and then apply the correct pulse amplitude or duration to the device, which inevitably increases the complexity of the peripheral circuitry design as well as the latency and energy consumption [67]. As an alternative way to improve the symmetry and linearity of the conductance response, lateral growth of the CF has been used [31,34]. However, this method leads to a lowered analog on/off ratio, which is an important parameter for high recognition accuracy [35,67,76,104,105].

Kim et al. reported improved conductance linearity and dynamic range in a Ag/SiN_x_/a-Si/p^++^ Si-based ECM device through Ge implantation [36]. Ge implantation induces structural defects in the bulk and surface regions of the a-Si layer, which enables spatially uniform Ag migration and nanocluster formation in the upper SiN_x_ layer, leading to enhanced synaptic behavior. Figure 5c,d show analog switching profiles for unimplanted and implanted devices, respectively. The dynamic range of the implanted device is higher than that of the unimplanted device for the overall pulse numbers. An increased current flow in an implanted a-Si layer can enhance Ag migration, which leads to increased Ag clustering in the SiN_x_ thin film and can improve the dynamic range of analog switching. In addition, the lower linearity in the depression of the unimplanted device, which has small-sized nanoclusters, is induced by the volatility characteristic, while the large-sized nanoclusters of the implanted device can improve the linearity for depression.

On the other hand, nanoclusters can form a CF with a curved shape in a Cu tip/SiO_2_/W cell, leading to a gradual increase in the current level, as reported by Yuan et al. [86]. Figure 5e,f show the gradual current change and the simultaneously obtained transmission electron microscope (TEM) image, respectively, where the diameter of the Cu tip close to the SiO_2_ is around 30 nm. With a bias sweep to +14 V, the device remains in the HRS (state I). A dark region corresponding to a Cu nanocluster suddenly appears when the bias is swept to +16 V. The existing Cu nanocluster starts to enlarge, which indicates that more Cu atoms migrate into the SiO_2_, and the device gradually switches to intermediate states (states II and III). In these states, the current gradually increases to the μA level. With an increase in bias, more and more nanoclusters emerge inside the oxide layer close to one another and gradually grow in a curved path toward the inert W electrode. When the sweep bias reaches +25 V, the nanoclusters finally form the shape of a curved CF with a diameter of 8 nm at the widest part. Simultaneously, the current reaches a high level, leading to set switching of the device to the LRS (state IV).

Choi et al. reported an improvement in conductance linearity, endurance, and device variability without a large loss in analog on/off ratio using CFs confined in one-dimensional dislocations of an epitaxial SiGe layer [35]. The defect-selective etching provides additional sites for Ag occupation along the pipeline for ion migration, which increases the analog on/off ratio to above 100, using the same pulse train conditions as those used for the unetched epitaxial SiGe layer with a low analog on/off ratio of 3. It was confirmed that securing enough space at dislocations also contributes to improving the linearity in the conductance response. The diameter of the dislocation is estimated to be few nanometers, which offers enough space to accommodate Ag ions. This technique of fabricating a pathway for cation movement can reduce the device variability with high endurance.

In order to implement large-scale neuromorphic systems, ECM devices should be incorporated into crossbar array structures. The main challenges for ECM devices in crossbar array structures are high on-state current, energy inefficiency, and overheads of the sensing circuits. In addition, there are non-negligible voltage drops across lines in the array, which lead to significant degradation of the dot-product computing precision. In order to reduce the on-state current of an ECM in a crossbar array, a one-transistor (1T)–one-resistor (1R) structure has been introduced. However, the introduction of a three-terminal transistor increases the energy and size cost. The formation of a tunnel barrier through an incomplete filament or stacking of a selector are better solutions to enhance the energy efficiency and scalability.

### 4.2. Anion-Movement-Based Filamentary Two-Terminal Synaptic Devices

The key advantages of anion-movement-based filamentary two-terminal synaptic devices include the compact structure, the CMOS compatibility, and the capability for hybrid or 3D integration, making them attractive for a broad range of applications including memory, analog devices, and reconfigurable circuits, as well as brain-inspired computing [37,38].

**Figure 6:**
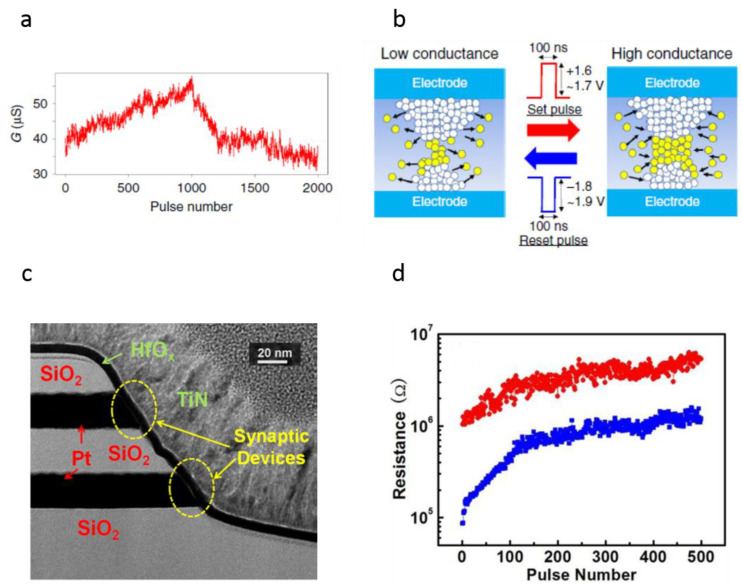
(**a**) Gradual increase (decrease) in conductance behavior in the HfO_2_-based resistive switching device induced by 1000 consecutive identical set (reset) pulses. (**b**) A conceptual schematic of a CF confined in the HfO_2_-based resistive switching device during switching corresponding to set and reset. Reprinted/adapted with permission from Ref. [41]. 2018, Springer Nature. (**c**) Cross-sectional TEM image of a 3D vertical structure based on HfO_x_ cells. (**d**) Synaptic behavior with different initial resistances (~100 kΩ and 1 MΩ) of the HfO_2_-based 3D vertical synaptic device. In the initial resistance of the 1 MΩ device, the energy consumption decreases below 1 pJ. Reprinted/adapted with permission from Ref. [42]. 2014, American Chemical Society.

Gong et al. implemented an anion-movement-based filamentary two-terminal synaptic device through a HfO_2_-based resistive switching device [41]. This device shows analog switching behavior, as shown in Figure 6a. The conductance of the device gradually increases and decreases under 1000 consecutive positive and negative pulses, respectively. The progressive change in conductance in the HfO_2_-based synaptic device is attributed to the modulation of the oxygen-vacancy-based filament by pulse stimulation. Figure 6b shows a schematic of its operation mechanism. The movement of the oxygen vacancies in response to an electrical signal has a probabilistic nature, which emerges as an inherent randomness in the conductance weight updates, superimposed on the expected signal.

Gao et al. developed a synaptic device based on a 3D vertical structure including several parallel oxide-based resistive switching devices on the same nanopillar, for the application of brain-inspired computing with high device density and low energy consumption [42]. Figure 6c shows a TEM image of a two-layer resistive switching device which has top and bottom HfO_x_-based cells on the same pillar. The analog switching behavior is obtained through only a gradual reset process to mimic biological depression. The abrupt set process is considered as positive feedback between the oxygen vacancy generation rate and the temperature/local field strength, while the gradual reset process is considered as negative feedback [37]. Figure 6d shows the gradual reset processes of the device, induced from two initial resistance states (~100 kΩ and 1 MΩ) by applying 500 consecutive negative identical pulses. For the case of an initial resistance state of ~1 MΩ, the maximum energy per spike drops to ~0.29 pJ. The reduced energy consumption is mainly attributed to the higher initial resistance of the device.

Yu et al. implemented an anion-movement-based filamentary two-terminal synaptic device with low energy consumption by stacking multiple layers of Pt/HfO_x_/TiO_x_/HfO_x_/TiO_x_/TiN [37]. The analog switching behavior is obtained through only a gradual reset process to mimic biological depression. The abrupt set process is considered as positive feedback between the oxygen vacancy generation rate and the temperature/local field strength, while the gradual reset process is considered as negative feedback. The gradual reset processes of five different devices were induced from two initial resistance states (~500 Ω and ~20 kΩ) by applying 400 consecutive negative identical pulses. A system-level simulation using these device-level experimental results revealed that the brain-inspired computing system may be robust against synaptic device variation. In addition, the synaptic device operates with very low energy consumption, which decreases from 24 pJ to 0.85 pJ as the initial resistance increases.

#### Challenges for Anion-Movement-Based Filamentary Two-Terminal Synaptic Devices

In an anion-movement-based filamentary two-terminal synaptic device, as in a cation-movement-based filamentary two-terminal synaptic device, the filament formation process and set switching is inherently abrupt and difficult to control. Hence, anion-movement-based filamentary two-terminal synaptic devices usually employ analog switching in the reset process, where the gradual rupture of CFs is involved [37,42]. However, in order that brain-inspired computing can be efficiently implemented using bidirectional analog switching of the synaptic device, the CF formation process should be controllable, leading to gradual set switching [40]. In addition, a large dynamic range is a critical parameter for high learning accuracy in brain-inspired computing and can provide improved tolerance to cycle-to-cycle and device-to-device variation [63].

**Figure 7:**
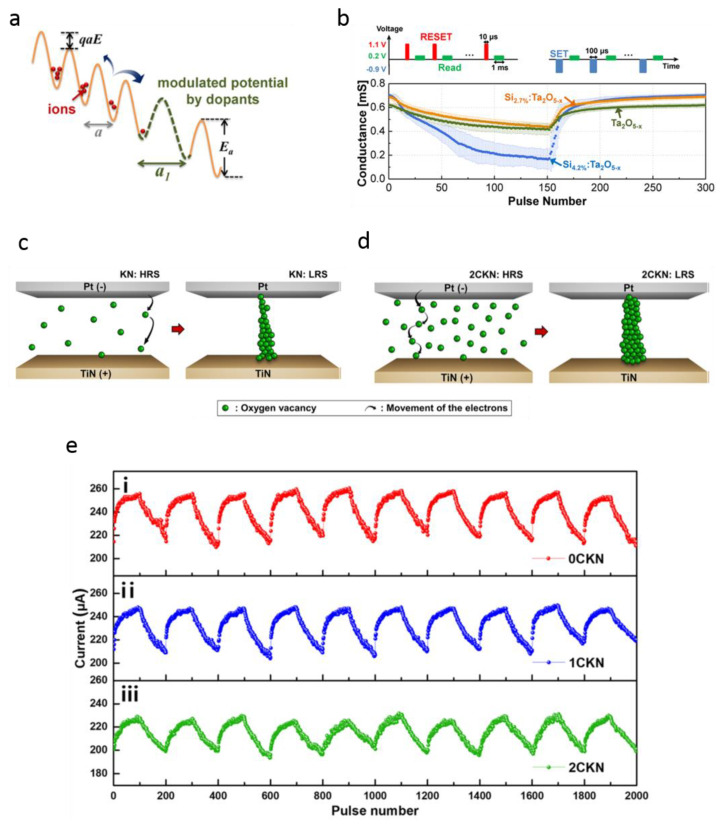
(**a**) Schematic illustration of nonlinear transport of different field−driven anions through the modulated hopping distances (a and $a_{1}$) for Ta_2_O_5-x_ layers with different Si doping concentrations. (**b**) The dynamic range of analog switching depending on the Ta_2_O_5-x_ layers with different Si doping concentrations under the same pulse train condition. The upward figure shows the consecutive reset and set pulse cycles applied to obtain depression and potentiation curves, respectively. Reprinted/adapted with permission from Ref. [39]. 2014, American Chemical Society. (**c**,**d**) Schematics of comparison of HRS and LRS in a Pt/KNbO_3_/TiN/Si with undoped (**c**) and Cu^2+^−doped (**d**) KNbO_3_ layers. (**e**) Potentiation–depression curves of Pt/KNbO_3_/TiN/Si with different Cu^2+^ doping concentrations ((i) 0.0, (ii) 1.0, and (iii) 2.0 mol %) in KNbO_3_ layer with a stimulation number of 100 for potentiation and depression, respectively. Reprinted/adapted with permission from Ref. [43]. 2020, American Chemical Society.

The fundamental atomic-level design of the active layer for switching can directly affect the transport of anions and optimize the switching characteristics. Kim et al. demonstrated that the dynamic range can be increased by controlling the ionic hopping parameters such as the hopping distance and drift velocity, based on atomic engineering of the active layer through doping with Si atoms [39]. Figure 7a shows a schematic of the potential energy landscape for the nonlinear transport of anions under a high electric field. The drift velocity of anions is strongly affected by the hopping distance a, which can be modulated by Si doping. Figure 7b exhibits the measured analog switching behaviors of Ta_2_O_5-x_ layers with different Si doping concentrations under pulse trains. It can be clearly observed that the dynamic range increases with the doping concentration of Si. Furthermore, an increased Si doping level results in a higher dynamic range but more digital-like switching, due to the larger hopping distance.

Park et al. reported that the growth/dissolution of CFs in a KNbO_3_ film is mainly influenced by the redox process of oxygen vacancies, and the linearity of synaptic weight updates in a Pt/KNbO_3_/TiN/Si synaptic device is improved when the number of oxygen vacancies in the KNbO3 switching layer is increased through doping with Cu^2+^ ions [43]. Figure 7c,d show schematic illustrations of HRS and LRS in synaptic devices with undoped and Cu^2+^-doped KNbO_3_ layers, respectively. The increased oxygen vacancies in the synaptic device with a Cu^2+^-doped KNbO_3_ layer cause the formation of CFs with increased radius during the LRS. Furthermore, it is demonstrated that an increase in the number of oxygen vacancies enhances both the effect of the redox process on the variation of the filaments and the linearity of the synaptic weight updates, as shown in Figure 7e. In addition, the potentiation–depression curves in the 2 mol % Cu^2+^-doped KNbO_3_ (2CKN)-based synaptic device are well maintained after 10 cycles, indicating that it has good potentiation–depression cycling properties.

VCM has great potential for applications at circuit or system levels due to fab-friendly materials and the Si-compatible low-temperature processing of the active layer. However, VCM has a high on-state current range, which can lead to energy inefficiency. In addition, the variability problem is yet to be solved. The variability in ECM cells is reduced through formation and modulation of the pathway for cations, while the variability in VCM is hard to solve, because valence is vulnerable to the ambient environment. In order to implement large-scale neuromorphic systems, it is required to develop VCM devices with low on-state current and variability.

### 4.3. Cation-Movement-Based Ferroelectric Two-Terminal Synaptic Devices

An ultra-thin ferroelectric material can act as a switching layer in cation-movement-based ferroelectric two-terminal resistive switching devices. The attractive advantages of ferroelectric two-terminal resistive switching devices such as fast switching speed and ultra-low power/energy consumption can lead to high performance in the synaptic devices based on them.

**Figure 8:**
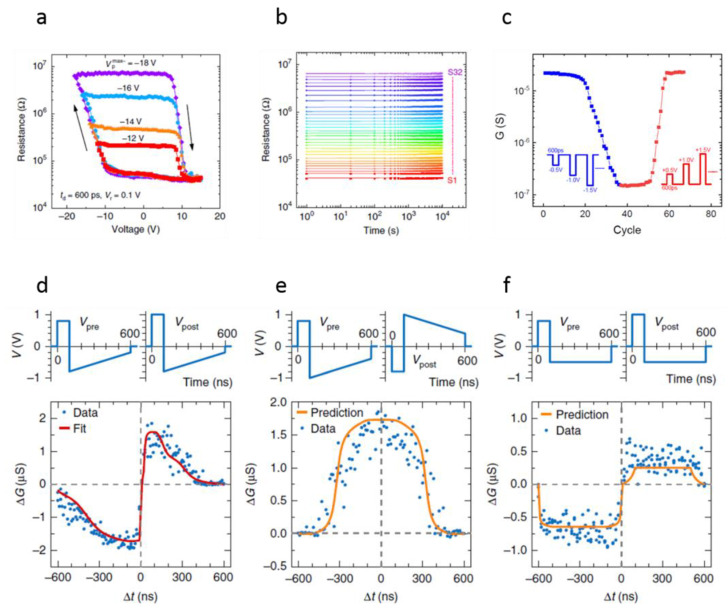
(**a**) Resistive switching with gradual change in Ag/BaTiO_3_ (~ 2.4 nm)/Nb:SrTiO_3_ ferroelectric tunnel junction under consecutive pulses (writing voltage = –18, –16, –14, and –12 V, fixed pulse duration = 600 ps). (**b**) Retention characteristics of the Ag/BaTiO_3_ (~ 2.4 nm)/Nb:SrTiO_3_ ferroelectric tunnel junction. (**c**) Analog switching behaviors of an ultra-thin ferroelectric-film-based synaptic device (Ag/BaTiO_3_ (~ 2.4 nm)/Nb:SrTiO_3_) under consecutive non-identical pulses with a sub-nanosecond (600 ps) duration. Reprinted/adapted with permission from Ref. [47]. 2020, Springer Nature. (**d**–**f**) STDP curves of different forms in an ultra-thin ferroelectric-film-based synaptic device (Co/BFO/CCMO). The top and bottom panels show presynaptic and postsynaptic spikes and the change in conductance, respectively. Reprinted/adapted with permission from Ref. [45]. 2017, Springer Nature.

Ma et al. reported that a synaptic device with an ultra-short switching time (600 ps) can be implemented by using a Ag/BaTiO_3_ (~2.4 nm)/Nb:SrTiO_3_ ferroelectric tunnel junction [47]. The upward and downward ferroelectric polarization directions correspond to the LRS and HRS, respectively. The switching between the two states is bipolar and, interestingly, not abrupt, leading to a broad range of intermediate resistance states. Figure 8a shows the gradual resistance versus voltage pulse behaviors obtained under different negative voltage ranges (−18, −16, −14, and −12 V) with a fixed pulse duration of 600 ps. The ultra-short switching time can be induced by both the high Nb concentration in the Nb:SrTiO_3_ semiconducting electrode and the low work function of the metal electrode. Figure 8b shows 32 distinct resistive states of the device which can be maintained with no degradation for 10^4^ s. To mimic biological synaptic plasticity, the sub-nanosecond (600 ps)-driven conductance change is measured, as shown in Figure 8c. A gradual change in the conductance similar to biological synaptic plasticity can be induced by increasing the amplitude of the negative or positive voltage pulses.

Boyn et al. reported that STDP with an operation time of a few hundred ns can be emulated through ferroelectric domain dynamics [45]. To mimic the various types of STDP shown in Figure 8d–f, they applied different pulse waveforms to a synaptic device based on ferroelectric switching. This procedure allows the generation of biologically realistic, accelerated (Figure 8d,e), or artificially designed (Figure 8f) STDP learning curves. In addition, Figure 8d–f show the excellent agreement between the conductance changes predicted using the nucleation-limited model of ferroelectric domains and the measured conductance variations associated with the various types of STDP.

Chanthbouala et al. reported that a ferroelectric synapse with ultra-short switching time (in the 10–200 ns range) can be implemented by using a Co (10 nm)/Au (10 nm)/BaTiO_3_ (2 nm)/La_0.67_Sr_0.33_MnO_3_ (30 nm) structure [44]. The upward and downward ferroelectric polarization directions correspond to the LRS and HRS, respectively. The switching between the two states is bipolar and, interestingly, not abrupt; a broad range of intermediate resistance states are observed. The nucleation and growth of ferroelectric domains by systematic variation of the pulse can lead to various resistance states, resulting in synaptic plasticity. Synaptic behaviors based on ferroelectric switching are demonstrated by applying consecutive pulses with a fixed amplitude.

#### Challenges for Cation-Movement-Based Ferroelectric Two-Terminal Synaptic Devices

A ferroelectric two-terminal synaptic device consists of an ultra-thin ferroelectric barrier inserted between two electrodes. It has attractive characteristics such as ultra-short switching time, nonvolatility, and ultra-low power/energy consumption. However, modulation of the barrier width in these synaptic devices is very limited, leading to a low dynamic range, although the tunneling transmittance depends exponentially on the barrier width [48,95]. The learning accuracy for the synaptic device dramatically decreases for a lower dynamic range [67,73,76]. In addition, to implement low-cost and large-scale arrays of synaptic devices based on ferroelectric switching, we must find suitable ferroelectric materials compatible with silicon or flexible substrates.

Wen et al. proposed a ferroelectric two-terminal resistive switching device employing a metal/ferro-electric/semiconductor heterostructure [95]. The potential energy profiles of the metal/ferroelectric/semiconductor for the LRS and HRS, respectively, depend on the ferroelectric polarization direction. Via polarization reversal in the ultra-thin ferroelectric barrier, the semiconductor surface can be switched between accumulation and depletion of the majority carriers as a result of a ferroelectric field effect. Hence, along with the switching of the barrier height in response to the polarization reversal in the ferroelectric barrier, additional tuning exists via the width of the barrier. The tunneling electrons experience an extra barrier over the space charge region if the semiconductor surface is depleted and the device is switched to the HRS. If the ferroelectric polarization points to the semiconductor, positive bound charges in the ferroelectric/semiconductor interface drive the n-type semiconductor surface into accumulation. This depolarization field lowers the barrier height and generates a higher tunneling transmittance, leading to the LRS. The semiconductor-based ferroelectric tunnel junction exhibits nonvolatile resistive switching behavior with a high dynamic range of above 10^4^.

**Figure 9:**
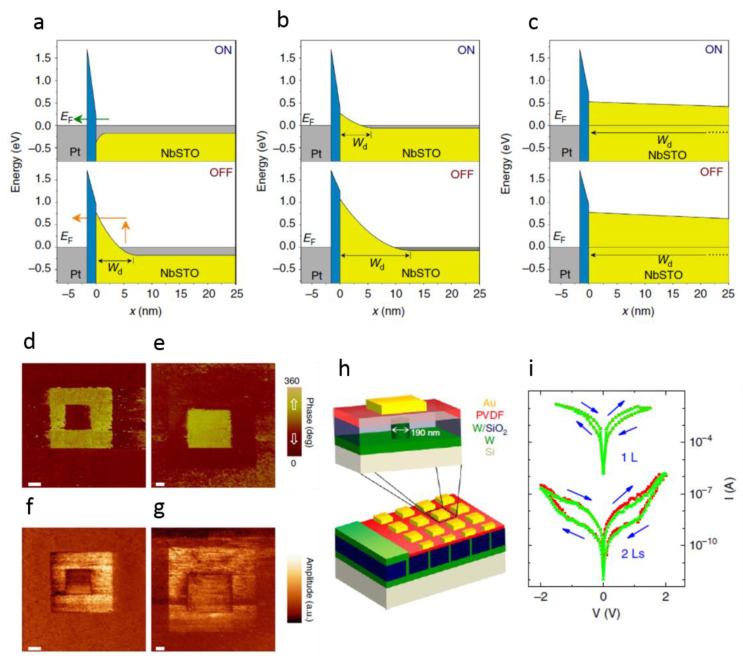
(**a**–**c**) Schematic illustration of potential energy profiles corresponding to the LRS and HRS in the Pt/BaTiO_3_/Nb:SrTiO_3_ structures with different Nb concentrations (1.0, 0.1, and 0.01 wt.%) in Nb:SrTiO_3_ semiconducting electrode. Reprinted/adapted with permission from Ref. [48]. 2017, Springer Nature. (**d**–**g**) PFM phase (**d**,**e**) and amplitude (**f**,**g**) images of bilayer (4.4 nm) and monolayer (2.2 nm) P(VDF-TrFE), respectively. (**h**) Schematics of the Au/ P(VDF-TrFE)/W ferroelectric tunnel-junction-based device. (**i**) Resistance switching behaviors in the 1- and 2-layer P(VDF-TrFE)-based ferroelectric tunnel junction devices. Reprinted/adapted with permission from Ref. [49]. 2016, Springer Nature.

Xi et al. proposed a ferroelectric two-terminal resistive switching device with a dynamic range of $6.0\times 10^{6}$ employing a Pt/BaTiO_3_/Nb:SrTiO_3_ heterostructure [48]. Figure 9a–c show the potential energy profiles of the Pt/BaTiO_3_/Nb:SrTiO_3_ in the LRS and HRS, respectively, which depend on the Nb concentration (1.0, 0.1, and 0.01 wt.%) in the Nb:SrTiO_3_ semiconducting electrode. For heavily doped Nb:SrTiO_3_ with a Nb concentration of 1.0 wt.%, the synaptic device shows direct electron tunneling through the ultra-thin BaTiO_3_ ferroelectric layer, leading to a large current in the LRS. When the Nb concentration decreases to 0.1 wt.%, a Schottky barrier in the BaTiO_3_/Nb:SrTiO_3_ appears, and thermally assisted tunneling is still pronounced, leading to a large LRS current comparable to direct tunneling. The device with a Nb concentration of 0.01 wt.% shows a low LRS current resulting from the suppression of thermally assisted tunneling, caused by a high and wide Schottky barrier. In the HRS, the device with a Nb concentration of 1.0 wt.% still shows a thermally assisted tunneling current due to the weak Schottky barrier, while the device with a Nb concentration of 0.1 wt.% has a Schottky barrier high and wide enough to suppress the thermally assisted tunneling. Therefore, the device with a Nb concentration of 0.1 wt.% can achieve a high dynamic range of $6.0\times 10^{6}$.

Tian et al. fabricated a resistive switching device based on an organic ferroelectric copolymer, poly(viny-lidene fluoride-trifluoroethylene) (P(VDF-TrFE)), which can be applied to large-scale arrays on silicon or flexible substrates [49]. Figure 9d–g show piezoresponse force microscopy (PFM) images of the out-of-plane ferroelectric polarization domains written on bilayer (4.4 nm) and monolayer (2.2 nm) P(VDF-TrFE), respectively. The bright (dark) PFM phase images in Figure 9d–e correspond to upward (downward) polarization after the writing process, indicating polarization reversal in bilayer and monolayer P(VDF-TrFE) via external bias. Figure 9h shows a schematic illustration of a resistive switching device with an ultra-thin P(VDF-TrFE) film fabricated on a Si substrate. The resistive switching behaviors between HRS and LRS were observed for devices with bilayer and monolayer P(VDF-TrFE), as shown in Figure 9i.

Recently, Li et al. demonstrated a cation-movement-based ferroelectric two-terminal synaptic device with high performance using a metal/ferroelectric/semiconductor heterostructure which has an extra depletion region on the semiconductor surface in the HRS [46]. The potentiation–depression curve for domain switching of the ferroelectric has a dynamic conductance range of 12–150 μS for 200 identical ultra-short pulses with a duration of 50 ns. The linearity of the potentiation–depression curve is the best among two-terminal synaptic devices.

Although FeRAM devices show good performances as synaptic devices, finding a Si-compatible process for the FeRAM is a big challenge. FeRAM devices with good performances usually consist of single-crystal or epitaxial ferroelectric materials. The high crystallinity requires high growth temperatures or special substrates. Hafnia-based ferroelectric materials show good performances, although they are grown at low temperatures by using atomic layer deposition [107]. In addition, a Si-compatible process for a hafnia-based ferroelectric has been demonstrated [108]. Therefore, a great deal of effort has been focused on realizing neural networks through a hafnia-based ferroelectric.

### 4.4. Ion-Movement-Based Electrochemical Three-Terminal Synaptic Devices

The ion-movement-based electrochemical three-terminal synaptic device operates in a fundamentally different way from filamentary and ferroelectric synaptic devices. It creates an ion diffusion profile at the surface of the channel, which can be better controlled than the stochastic formation/rupture of CFs and can be more robust to cycle-to-cycle and device-to-device variations [61,96,109].

Burgt et al. fabricated a three-terminal device which comprises a postsynaptic electrode, a poly(3,4-ethylene-dioxythiophene):polystyrene sulfonate (PEDOT:PSS) film partially reduced with poly(ethylenimine) (PEI), interfaced with a PEDOT:PSS presynaptic electrode, and an electrolyte (Nafion) [61]. The potentiation and depression can be induced by a series of 500 pulses with ultra-low amplitude (~1 mV), resulting in 500 distinct conductance states. In addition, this device can be operated by injecting a presynaptic current pulse, displaying a nearly perfect linearity and low cycle-to-cycle variability. The developed flexible device has the potential for low-cost fabrication of arrays, which enables the integration of on-board neuromorphic computing and learning in implantable prosthetics, neural electrode arrays, or any other flexible large-area electronic system.

**Figure 10:**
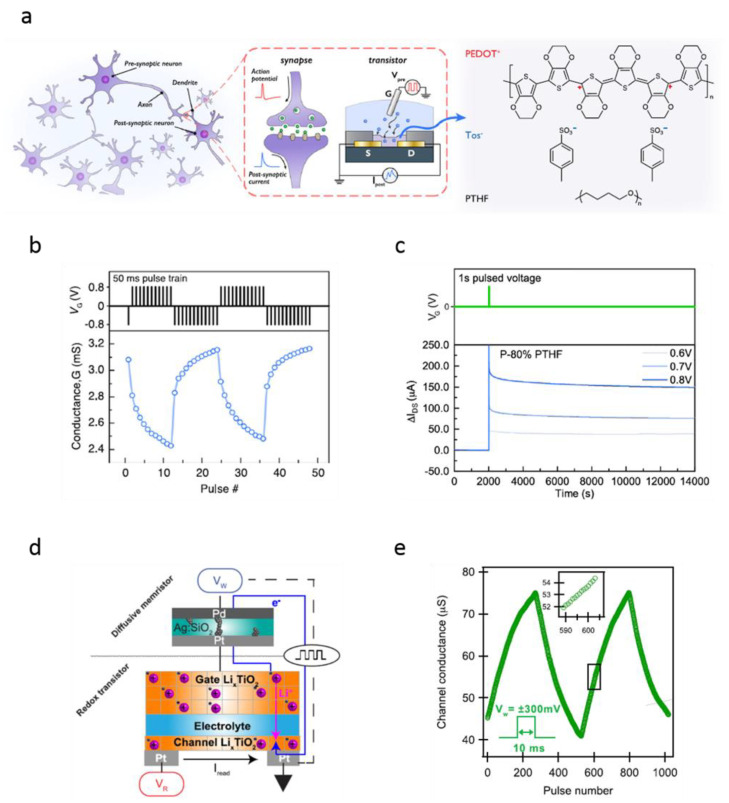
(**a**) Biological synapses and ECRAM–based synaptic device and the chemical structures of PEDOT^+^, Tos^−^, and PTHF. (**b**) Potentiation–depression curves of an ECRAM–based synaptic device with PEDOT:Tos/P–80 % PTHF as the active channel. These curves show 12 discrete states by injecting a writing voltage with ultra-low amplitude (+0.8 and −0.8 V). (**c**) Nonvolatile characteristic of the ECRAM-based synaptic device with PEDOT:Tos/P–80 % PTHF as the active channel under pulse stimulation with different amplitudes (0.6, 0.7, and 0.8 V). Reprinted/adapted with permission from Ref. [54]. 2021, Springer Nature. (**d**) Schematic illustration of a composite system of an inorganic redox transistor and a diffusive memristor. (**e**) Potentiation–depression curves of a composite system of an inorganic redox transistor and a diffusive memristor displaying nearly perfect linearity and low cycle-to-cycle variability under an injected pulse train with a fixed ±300 mV amplitude and 10 ms duration. Reprinted/adapted with permission from Ref. [55]. 2019, American Chemical Society.

Ji et al. fabricated an electrochemical three-terminal synaptic device employing a poly(3,4-ethylene-dioxythiophene):tosylate (PEDOT:Tos)/polytetrahydrofu-ran (PTHF) composite as the active channel [65]. Figure 10a shows schematics of the electrochemical three-terminal synaptic device corresponding to a biological synapse and the chemical structures of PEDOT^+^, Tos^-^, and PTHF. The PEDOT:Tos/PTHF composite, as the active channel, is fabricated by the vapor-phase polymerization method. Different mass ratios of PTHF are adopted, and the resulting films are annotated as P-x% PTHF (x = 0, 20, 50, 80, 90) (in the film, x% is the PTHF by mass). The electrochemical three-terminal synaptic device with P-80% PTHF reveals potentiation–depression behaviors with discrete resistance states which are induced by a gating pulse train with a low voltage (<0.8 V) and are maintained over a long time (>200 min), as shown in Figure 10b,c.

Li et al. reported that an inorganic electrochemical three-terminal synaptic device with a Li_x_TiO_2_ diffusive memristor displays high linearity at both low current level and low writing pulse amplitude [66]. Moreover, the developed device does not need a read-select metal oxide semiconductor (MOS) transistor to switch the device between reading and writing operations. Figure 10d shows a schematic illustration of the inorganic electrochemical three-terminal synaptic device consisting of an inorganic redox transistor and a diffusive memristor selector. To check the conductance updates, a pulse train with a fixed amplitude of ±300 mV and 10 ms duration was applied to the device. Figure 10e shows the gradual change in the device conductance, with high linearity, symmetry, and a precision of 250 with an identical pulse train.

α-MoO_3_ is a layered 2D material which allows the reversible intercalation of cations (e.g., H+ and Li+), via a Faradaic reaction involving the reduction/oxidation of Mo ions [51]. Yang et al. reported an all-solid-state electrochemical transistor consisting of a Li-ion-based solid dielectric and 2D α-MoO_3_ nanosheets as the channel [51]. The devices can achieve nonvolatile conductance modulation through reversible intercalation of Li ions into the α-MoO_3_ lattice. The channel conductance shows nonvolatile characteristics, where each channel conductance obtained after pulse stimulation decays to a stable value different from the previous one. To check the linearity, the asymmetric ratio (AR) was calculated from the 50-cycle LTP and LTD results. The obtained AR value was 0.31 ± 0.12, which is well below those of two-terminal memristive devices triggered by identical pulses (0.55–0.88). In addition, potentiation–depression curves were measured for 15 electrochemical transistors with a device-to-device variation of <12%, indicating good device-to-device uniformity.

#### Challenges for Ion-Movement-Based Electrochemical Three-Terminal Synaptic Devices

Electrochemical three-terminal synaptic devices can induce better modulation of the conductance than two-terminal synaptic devices, due to the separation of the read and write processes. The major challenge preventing the scale-up of inorganic electrochemical three-terminal synaptic devices is the utilization of Li as the doping ion. Li is not compatible with current CMOS processing due to its high volatility and concerns over manufacturing tool contamination [67,68]. In addition, the organic nature of the active channel material in organic electrochemical three-terminal synaptic devices presents challenges for Si-compatible device fabrication and long-term stability [68].

**Figure 11:**
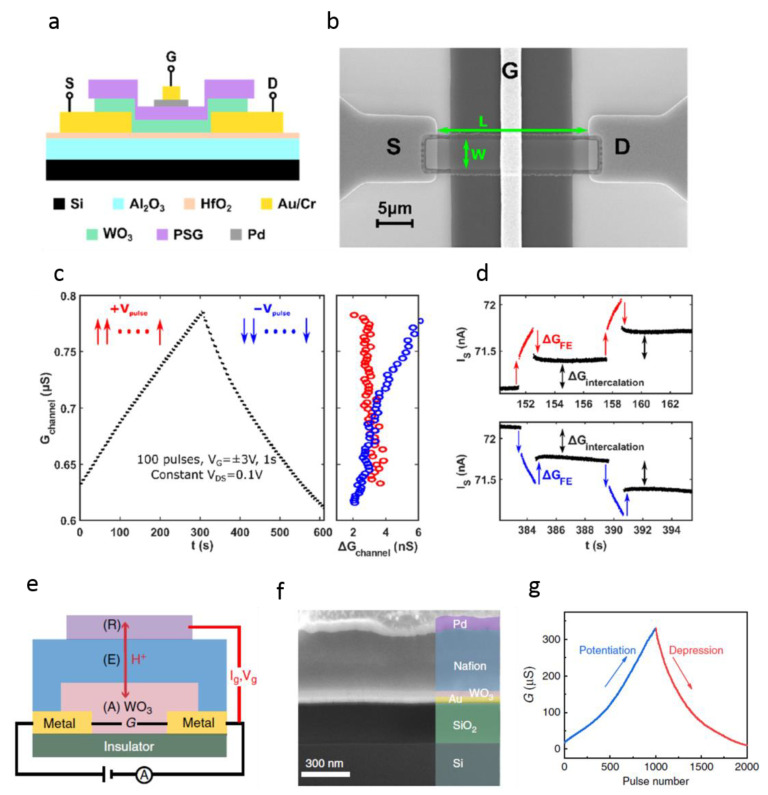
(**a**) Schematic illustration of the protonic programmable resistor for CMOS–compatible electrochemical three–terminal synaptic device. (**b**) The top–view scanning electron microscope image of an electrochemical three–terminal synaptic device with phosphosilicate glass as an electrolyte layer. (**c**) Potentiation–depression curve of a CMOS–compatible electrochemical three–terminal synaptic device consisting of phosphosilicate glass as an electrolyte layer obtained by injecting 100 consecutive pulses with ±3 V amplitude and 1s duration. This curve shows high linearity and low cycle–to–cycle variability. (**d**) The current response of the channel for multiple discrete currents with nonvolatile behavior in a CMOS–compatible electrochemical three–terminal synaptic device with phosphosilicate glass. Reprinted/adapted with permission from Ref. [56]. 2021, American Chemical Society. (**e**,**f**) Cross–sectional schematic illustration (**e**) and scanning electron microscopy image (**f**) of an inorganic electrochemical three–terminal synaptic device based on proton intercalation in a WO_x_ channel. (**g**) Potentiation–depression curves of an inorganic electrochemical three–terminal synaptic device with the proton–conductive solid–polymer electrolyte (Nafion–117) as electrolyte, via operation spikes of ±200 nA amplitude with 5 ms duration. The curves reveal high linearity, a large dynamic range, and high precision. Reprinted/adapted with permission from Ref. [57]. 2020, Springer Nature.

Onen et al. fabricated an electrochemical three-terminal synaptic device using a CMOS-compatible nonvolatile protonic programmable resistor enabled by the phosphosilicate glass electrolyte layer [67]. Figure 11a,b show the schematic illustration and the top-view scanning electron microscope image of the device, respectively. To mimic biological synaptic plasticity, the pulse stimulation is applied to the gate of the developed electrochemical three-terminal synaptic device for the writing process. The conductance of the channel in the device is modulated with high linearity during the writing process, with 100 consecutive pulses with ±3 V amplitude and 1s duration, as shown in Figure 11c. In addition, the current response of the channel as a function of time, shown in Figure 11d, shows switching between multiple discrete current levels during potentiation and depression processes, as well as good retention during the read operation without gate pulses.

Yao et al. demonstrated an inorganic electrochemical three-terminal synaptic device based on proton intercalation instead of Li-ion movement in a WO_x_ channel, which is CMOS-compatible [68]. The synaptic device consists of a source (Au), drain (Au), gate (Pd), proton-conductive solid-polymer electrolyte (Nafion-117), and WO_x_ channel, as shown in Figure 11e. The Pd gate electrode serves the dual role of a reservoir of protons and electrons. After the deposition of Pd, hydrogen is introduced to form PdH_x_. The electrolyte has a thickness of 300–400 nm and is fabricated by a spin-coating method (Figure 11f). The device is operated using positive or negative gate-current pulses which are applied in order to migrate protons into or out of the WO_x_ channel, respectively. Under positive current pulses, PdH_x_ is electrochemically oxidized, releasing protons that can move through the Nafion-117 electrolyte and intercalate into the WO_x_. As a result, the conductance of the WO_x_ channel is gradually increased, similarly to the potentiation of biological synapses, as shown in Figure 11g. On the other hand, the conductance of the channel is decreased due to the release of protons from the channel during the application of negative current pulses to the gate electrode, leading to depression. In addition, the developed synaptic device shows high linearity, a large dynamic range, and high precision.

Organic electrochemical three-terminal synaptic devices suffer from a limited dynamic range (<2×) between the highest and lowest conductance states induced using short (~1 μs or less) write pulses [63]. The intrinsic semiconducting polymer poly(2-(3,3-bis(2-(2-(2-methoxyethoxy)ethoxy)ethoxy)-[2,2-bithiophene]-5-yl)thieno[3,2-*b*]thiphene) [p(g2T-TT)] was introduced to improve the performances of organic electrochemical transistors [110]. Melianas et al. used ion gel electrolytes by mixing the polymeric insulator poly (vinylidene fluoride-co-hexafluoropropylene) (PVDF-HFP) with common ionic liquids such as 1-ethyl-3-methylimidazolium bis (trifluoromethylsulfonyl)imide (EMIM:TFSI) or 1-ethylimidazolium bis(trifluoromethylsul-fonyl)imide (EIM:TFSI).44. The p(g2T-TT) channel device enables a higher dynamic range (~4×) even under considerably shorter (300 ns) write pulses with an amplitude of 1 V than those needed for PEDOT:PSS channel devices.

ECRAM was recently developed, motivated by a battery system. Therefore, the development of device-level ECRAM presents an open field of research into tasks such as searching for materials, developing device architectures, and optimizing processes. Recently, ECRAM devices fabricated with a Si-compatible process have shown significant potential as synaptic devices. It is expected that large neural networks based on ECRAM produced using the Si-compatible process will be investigated.

## 5. Conclusions

The development of synaptic devices for brain-inspired computing has progressed in the last few years. In this review, we discussed the fact that ion-movement-based synaptic devices of different types can be used to realize brain-inspired computing. In addition, we reviewed the strengths and weaknesses of each of these synaptic devices when applied to ANNs in brain-inspired computing. However, each device has remaining issues to be resolved for fully satisfying the specifications of a synaptic device for ANN: (1) linearity and cycle-to-cycle variation for a filamentary system based on cation and anion movement; (2) linearity and device-to-device variation for a cation-movement-based ferroelectric; and (3) dynamic range for an ion-movement-based electrochemical three-terminal device. To address these issues, various fields must be involved, from materials science to device design. In addition, synaptic devices should be optimized using circuit- or system-level simulators based on a compact model of reliability degradation for performance benchmarking and circuit design. If the challenges for each of the different types of synaptic devices are resolved, large-scale integration based on ion-movement-based synaptic devices will be realized in the near future, leading to the implementation of brain-inspired computing chips with low energy consumption, high scalability, and high processing speed. Therefore, ion-movement-based synaptic devices for brain-inspired computing continue to represent a potentially attractive solution to the problem of highly parallel and distributed processing of massive amounts of data, and thus can be expected to remain an active area of research.

## Data Availability

Not applicable.
